# Google search spike of “My Eyes Hurt” in United States after solar eclipse: an analysis and future prevention

**DOI:** 10.1038/s41433-024-03145-7

**Published:** 2024-05-21

**Authors:** Ethan Waisberg, Joshua Ong, Andrew G. Lee

**Affiliations:** 1 Department of Ophthalmology, University of Cambridge, Cambridge, UK; 2 Department of Ophthalmology and Visual Sciences, University of Michigan Kellogg Eye Center, Ann Arbor, MI USA; 3 Center for Space Medicine, Baylor College of Medicine, Houston, TX USA; 4 Department of Ophthalmology, Blanton Eye Institute, Houston Methodist Hospital, Houston, TX USA; 5 The Houston Methodist Research Institute, Houston Methodist Hospital, Houston, TX USA; 6 Departments of Ophthalmology, , Neurology, and Neurosurgery, Weill Cornell Medicine, New York, NY USA; 7 Department of Ophthalmology, University of Texas Medical Branch, Galveston, TX USA; 8 University of Texas MD Anderson Cancer Center, Houston, TX USA; 9 Texas A&M College of Medicine, Bryan, TX USA; 10 Department of Ophthalmology, The University of Iowa Hospitals and Clinics, Iowa City, IA USA

**Keywords:** Scientific community, Risk factors

Solar eclipses are a rare celestial event that occurs when the Moon obstructs the Sun, and have enthralled humanity for hundreds of years. Since the time of Plato, the negative effects of sun viewing have been well known, and Socrates even advised that an eclipse only be watched indirectly, through a reflection in water [1]. However the allure of witnessing this rare celestial event can lead individuals to potentially disregard safety advice, and directly look at the eclipse. Although our team typically focuses on reducing the ophthalmic risks faced by astronauts during long duration spaceflight [2–4], in this paper we discuss how to reduce the risks from direct observation of solar eclipses on Earth and emphasize protective measures.

On April 8, 2024, the day of the total solar eclipse, “my eyes hurt” became a trending Google search result at approximately 3 pm Eastern Time in the United States (Fig. 1). This timing coincided with nearly the exact time that the total solar eclipse began. The various subregions that also searched this term the most, including states such as Vermont, Arkansas, Michigan, Ohio and Indiana were directly along the path of the eclipse. Publicly available Google search trend data has previously been shown as a useful tool for accurate estimation of public health issues, such as influenza epidemics [5]. Using Google Trends to examine search data is particularly appealing as this data can be seen in real-time and can show relative search volume on a scale between 0 and 100, based on chosen timeframes.

**Fig. 1:**
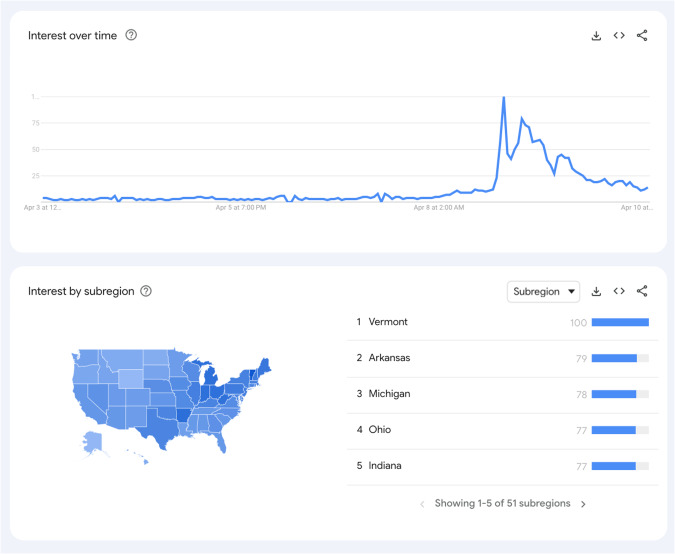
Google trends for “my eyes hurt” on April 8, 2024.

## Eclipse retinopathy

Solar retinopathy is a well-known clinical phenomena involving retinal damage as a result of viewing the sun. Symptoms typically include photosensitivity, blurred vision, headache and various patterns of scotoma [6]. Eclipse retinopathy, refers to a solar retinopathy resulting from viewing a solar eclipse. Michaelides et al. [7] conducted the largest nationwide study of the visual effects in the United Kingdom following a full solar eclipse in 1999. This study found that 70 individuals reported cases of visual loss following the eclipse, and half of these cases presented within 2 days of the occurrences of the eclipse. Although 84% of these patients had an abnormal macular appearance at presentation, none of the cases experienced continued vision loss after 6 months [7]. In another cohort of 20 patients that became symptomatic from watching this same eclipse, 3 of these patients had an unresolved central scotoma after 7 months, which eventually resolved at 21 months follow up [8]. A central scotoma is particularly challenging for affected patients as central vision is crucial for tasks such as reading, facial recognition, depth perception and driving ability [9–11].

Thanos et al. [12] found that albino rats exposed to a partial (90%) solar eclipse resulted in neuronal apoptosis. Dying retinal cells were seen at 24 hours after exposure reaching a peak at 6 days after exposure. This was the first animal study showing the cellular effects of eclipse retinopathy, which includes glio-vascular responses and neuronal apoptosis [12]. Although at the time of writing, there are currently no proven methods to treat solar retinopathy, prevention through patient education to avoid directly looking at solar eclipses is the key to avoid this condition.

## Protective measures

Effective protective measures can be used to safely view an eclipse. Indirect observation methods such as a pinhole projector or through a solar telescope with specialized lenses would be the safest methods of viewing an eclipse. Wearing eclipse glasses or solar viewing cards to view a solar eclipse, although commonly recommended, can have potential problems. The National Aeronautics and Space Administration (NASA) has recommended that solar viewers should comply with the ISO 12312-2 International Standard [13]. Substandard, or counterfeit glasses may not effectively block harmful radiation from the Sun and put individuals at a risk of severe eye damage. Additionally, small scratches in lenses could also compromise the ability of the lenses to block solar radiation and lead to eye injuries.

Solar eclipses pose a danger to ocular health when observed directly without adequate retinal protection. Increased public awareness is essential to further mitigate these risks through collaborative efforts with both public health agencies and eye care specialists.
